# Binary mixtures of imidacloprid and thiamethoxam do not appear to cause additive toxicity in fathead minnow larvae (*Pimephales promelas*)

**DOI:** 10.3389/ftox.2023.1282817

**Published:** 2023-11-20

**Authors:** Anya J. Jeninga, Nicole Kooij, Elisabeth Harrahy, Tisha C. King-Heiden

**Affiliations:** ^1^ Department of Biology and River Studies Center, University of Wisconsin-La Crosse, La Crosse, WI, United States; ^2^ Department of Biological Sciences, University of Wisconsin-Whitewater, La Crosse, WI, United States

**Keywords:** zebrafish, neonicotinoid, behavior, mixture, imidacloprid, thiamethoxam

## Abstract

**Introduction:** Considerable use of neonicotinoid insecticides has resulted in their detection in surface waters globally, with imidacloprid (IM) and thiamethoxam (TM) frequently found together. Neonicotinoids are selective agonists for invertebrate nicotinic acetylcholine receptors (nAChR) leading to paralysis and death. While not overtly toxic to vertebrates, growing evidence suggests that chronic exposure to individual neonicotinoids can cause adverse health effects in fish. This work examined whether chronic exposure to binary mixtures of imidacloprid (IM) and thiamethoxam (TM) would be more toxic to fathead minnow (*Pimephales promelas*) larvae than either insecticide alone.

**Materials and Methods:** Embryos were exposed to a 1:1 mixture of IM and TM (0.2, 2, 20, 200 or 2,000 μg/L of each pesticide) or a 1:5, 1:10, or 1:20 mixture of IM and TM (0.02 μg/L of IM with 0.1, 0.2, or 0.4 μg/L of TM) for a total of 8 days. Survival, developmental toxicity, embryonic motor activity, and startle escape responses were quantified.

**Results:** Survival and growth were reduced, and hatching induced by exposure to a 1:1 mixture containing > 2 μg/L of each insecticide, but not following exposure to mixtures containing environmentally-relevant concentrations. Acute exposure to a 1:1 mixture did not alter embryonic motor activity; however, chronic exposure to these mixtures resulted in a slight but significant decrease in embryonic movements. Only 1:1 mixtures of high concentrations of IM and TM altered the startle escape response by increasing latency of response; however, a significant proportion of fish exposed to 1:1 mixtures had altered latency and burst speed. Taken together, these behavioral indicators of nAChR activation suggest that in mixtures, neonicotinoids could interfere with nAChR signaling despite their low affinity for the nAChR.

**Conclusion:** Our findings suggest that toxicity of binary mixtures of IM and TM is primarily driven by IM, and that mixtures of IM with TM do not appear to cause significant additive toxicity when compared with our previous studies evaluating each neonicotinoid alone. Given the limited toxicological data available for mixtures of neonicotinoid insecticides in fish, further study is required to better understand the ecological risks these insecticides may pose to aquatic ecosystems.

## 1 Introduction

Insecticides in the neonicotinoids class are among the most widely used, making up approximately 80% of seed treatments sold worldwide (Byrne and Toscano, 2006; Jeschke et al., 2011). Due to their frequent application and high water solubility, neonicotinoid insecticides have become pseudo-persistent in streams and groundwater (Huseth and Groves, 2014; Anderson et al., 2015a; Hladik and Kolpin, 2016; Thompson, 2020). The first-generation neonicotinoid, imidacloprid (IM), has been widely used since the 1980s (Abou-Donia et al., 2008), and thiamethoxam (TM) is a second-generation neonicotinoid also used world-wide since the late 1990s (Simon-Delso et al., 2015). Global monitoring programs have detected both neonicotinoids in surface waters, with IM and TM being frequently detected together (Hladik and Kolpin, 2016; DATCP, 2018; Berens et al., 2021). In Wisconsin (U.S.A), the presence of these neonicotinoid insecticides in various surface and drinking water sources has raised concerns, with IM detected in surface waters in the range 0.05–0.08 µg IM/L and TM detected in surface waters at 0.057–2.78 μg TM/L (DATCP, 2018). Their high specificity for the invertebrate nicotinic acetylcholine receptor (nAChR) was thought to limit their effects on vertebrates (Yamamoto et al., 1998); however, several studies demonstrate their toxicity in fish (Beggel et al., 2012; Gibbons et al., 2015; Thompson, 2020; Victoria et al., 2022a; Victoria et al., 2022b). Taken together, this suggests that fish, and aquatic ecosystems in general, may be at risk from exposure to mixtures of these insecticides (Anderson et al., 2015b; Sánchez-Bayo et al., 2016).

Since neonicotinoid insecticides occur in aquatic ecosystems as mixtures, it is important to compare adverse effects following exposure to mixtures of neonicotinoids with those following exposure to individual neonicotinoids (Weisner et al., 2021; Schmidt et al., 2022; Stehle et al., 2023). Environmental contaminants that share a common mode of action can have similar toxicokinetics and toxicodynamics, and exposure to mixtures of such contaminants can lead to additive, synergistic or antagonistic toxicity (Deneer, 2000; Carvalho et al., 2014; Cedergreen, 2014). Laboratory studies examining the toxicity of mixtures of neonicotinoids in aquatic invertebrates suggest that they may cause additive or synergistic toxicity, but the toxicity of the mixture depends on which neonicotinoids are present and at what relative concentrations (Lanteigne et al., 2015; Maloney et al., 2017; Maloney et al., 2018), and that observed toxicity of a mixture is sometimes dominated by one particular neonicotinoid (Macaulay et al., 2021; Schmidt et al., 2022). Few studies have evaluated the toxicity of mixtures of neonicotinoid insecticides in fish. Given their lower affinity for the nAChR in fish (Yamamoto et al., 1998; Tomizawa and Casida, 2005), mixtures of neonicotinoids may or may not behave similarly in fish as they do in invertebrates. Binary mixtures of higher concentrations of IM (66 mg/L) and the neonicotinoid clothianidin (30 mg/L) cause additive neurotoxicity and oxidative stress to fingerling rohu fish (*Labeo rohita*) (Gajula et al., 2023), while a binary mixture of 33 and 15 mg/L of IM and clothianidin, respectively, inhibited electrolyte and ATPase activity at the gill of fingerling rohu fish (Veedu et al., 2022; Kumar and Das Vanamala, 2023). As a mixture, thiamethoxam and the neonicotinoid acetamiprid did not increase toxicity in observed endpoints, which when exposed individually caused oxidative stress and altered biochemical responses (Veedu et al., 2022). In all of these studies, only one or two mixture concentrations were tested; therefore, it is not possible to discern whether a binary mixture of these neonicotinoids would lead to additive, synergistic, or antagonistic toxicity. Further complicating this work is the fact that fish are relatively insensitive to these insecticides. Since the LC50s in fish tend to be at concentrations (mg/L) that are substantially higher than what are found in the environment (µg or ng/L), use of toxic unit concepts or concentration-addition null models are not relevant to the concentrations of neonicotinoid pesticides to which wild fish populations are exposed. To the best of our knowledge, no studies have used embryo larval exposure studies to evaluate the toxicity of neonicotinoid mixtures, or included mixtures at concentrations that are environmentally relevant.

Our previous work showed that individually, IM and TM cause subtle toxicity in fathead minnow larvae (Victoria et al., 2022a; Jeninga et al., 2023). Here, we used fathead minnow (*Pimephales promelas*) to test the hypothesis that embryo larval exposure to binary mixtures of IM and TM that include environmentally relevant concentrations would be more toxic than exposure to either pesticide alone. In an effort to reduce animal usage, we did not incorporate or repeat exposures to IM or TM individually for this study, but given the similarity in experimental design, can generally compare results across the three studies. Since pesticides that have the same mode of action can cause additive toxicity (Belden et al., 2007; Howard and Webster, 2009; Sigurnjak Bureš et al., 2021), we hypothesized that a 1:1 mixture of IM and TM would cause additive toxicity by further reducing survival and impacts on behavioral endpoints associated with activation of the nAChR. Since IM appears to be more toxic than TM to fathead minnow larvae (Victoria et al., 2022a; Jeninga et al., 2023), we also examined the potential for increased toxicity when fish were exposed to IM at a concentration not likely to cause toxicity (0.05 µgIM/L) in combination with TM at environmentally-relevant ratios found in Wisconsin surface waters (1:5, 1:10, and 1:20 IM:TM) (DATCP, 2018). Developmental toxicity was characterized by evaluating hatching, growth, signs of abnormal development, and survival. Both IM and TM are capable of binding to the nAChR, the activation of which coordinates behavioral movements and neuromuscular activity. Chronic exposure to environmentally relevant concentrations of both IM and TM individually suppress the expression of several locomotor-related genes associated with behavioral functions in zebrafish (Zhang et al., 2023). Therefore, we also evaluated the ability of co-exposure to IM and TM to alter embryonic motor activity and the predator escape response.

## 2 Materials and Methods

### 2.1 Chemicals and test species

Thiamethoxam (TM, >99% purity) and imidacloprid (IM, >99% purity) were purchased from Sigma-Aldrich, Inc. Solvent free TM and IM dosing solutions were prepared through serial dilutions of 20,000 μg/L stock solutions with moderately hard water fathead minnow water (80–100 mg/L CaCO_3_, pH 7.4–7.8; US EPA, 2002). Binary mixtures were prepared fresh using a fixed ratio design targeting 1:1 ratios of IM:TM at concentrations of each pesticide previously shown to cause minor toxicity in fish (Victoria et al., 2022a; Jeninga et al., 2023), as well as ratios that represent observed low detection environmental concentrations for IM with increasing environmentally-relevant concentrations of TM of 1:5, 1:10, and 1:20 of IM:TM. Fresh dosing solutions sufficient for the 8 days exposure were made for each experiment by diluting stock samples into moderately hard fathead minnow water. Between solution exchanges, dosing solutions were stored at 4°C in amber vials and brought to room temperature prior to solution exchange. One sample from each dosing solution (1,470 µL) were collected just after preparation and at the end of the experiment, combined with an internal standard (15 µL deuterated 1 μg/mL IM and 15 µL deuterated 1 μg/ml TM) and stored in amber autosampler vials at −20°C until analysis (*n* = 6 samples for each exposure ratio were measured). Concentrations of IM and TM were confirmed using high-performance liquid chromatography with tandem mass spectrometry (HPLC/MS/MS) by the Lumigen Instrument Center at Wayne State University (Detroit, MI, United States); the relative standard deviation was 12% and 18% for IM and TM, respectively. Limit of detection was 2.8 ng IM/L and 3.8 ng TM/L. The spike recovery was 99% and 94% for IM and TM, respectively. The LOQ was 3 ng IM/L and 2 ng TM/L. Table 1 summarizes confirmed concentrations for all mixtures (*n* = 6).

**TABLE 1:** Summary of confirmed concentrations for Imidacloprid (IM) and Thiamethoxam (TM) used in binary mixture studies. Confirmed concentrations had a 12% and 18% relative standard deviation among samples tested for IM and TM, respectively (*n* = 6 for each mixture).

Ratio IM:TM	IM (µg/L)	TM (µg/L)
1:1	0.36	0.52
1:1	1.8	1.9
1:1	23.6	21.2
1:1	213	204
1:1	2,245	2,590
1:5	0.03	0.14
1:10	0.03	0.39
1:20	0.03	0.68
LOD	0.0028	0.0038
LOQ	0.003	0.002
Spike recovery	99%	94%

All experiments were approved by the Institutional Animal Care and Use Committee (IACUC) Animal Use Protocol (Protocol 3–19) and toxicity tests followed standard protocols outlined by the US EPA and the Organization for Economic Co-operation and Development (US EPA, 2002; OECD, 2012). Fathead minnow eggs were provided by the Wisconsin State Laboratory of Hygiene (Madison, WI). Embryos were removed from spawn tiles shortly after fertilization, pooled and randomly sorted by under a dissecting microscope into exposure groups.

For all experiments, exposures were completed at 25°C with a 16-h light cycle. Water quality parameters (pH, temperature, DO) were assumed not changed within dosing solutions since they were made fresh for each experiment and 100% of dosing solutions were exchanged daily.

### 2.2 Potential for acute exposure to a 1:1 mixture of IM and TM to stimulate behavioral indicators of nAChR activation

Acute exposure to nicotine in zebrafish larvae (*Danio rerio*) actives the nAChR, causing increases in spontaneous contractions of the tail (embryonic motor activity) until overstimulated, resulting in paralysis (Victoria et al., 2022a). In contrast, previous studies show that individually, neither IM nor TM have sufficient binding affinity to alter embryonic motor activity or cause paralysis in fathead minnows following acute exposure (Victoria et al., 2022a; Jeninga et al., 2023). In the current study, we tested the hypothesis that acute exposure to a mixture of IM and TM would act in an additive manner to alter embryonic motor activity. Eggs were collected from 3 different spawn groups (one male and two females), pooled, and were randomly sorted so that there were 10 embryos in each well of a 24 well plate (*n* = 8 replicate wells per mixture). At 1 day post fertilization, embryos were dosed with the appropriate concentrations of IM and TM to produce the target dosing solutions for all mixtures (1:1 mixture of 0, 2, 20, 200 or 200 μg/L). Following 1 min of exposure, the embryonic motor activity was assessed by quantifying the frequency of spontaneous contractions of the body using a dissecting microscope attached to a Nikon 80i camera (Nikon Instruments, Inc., Mellville, NY, United States). A 1-min video of all embryos in each exposure group was recorded at 43 frames per second (fps) using NIS Elements software (Nikon Instruments, Inc., Mellville, NY, United States) in a cycled pattern by to eliminate time as a source of variability. The number of tail bends/min for all fish within a given replicate exposure group was averaged before statistical analysis to avoid pseudo-replication.

### 2.3 Effects of chronic exposure to IM and TM mixtures on survival, hatching and growth

Embryos (∼6 h post-fertilization) were randomly placed into wells of a 24-well plate, with 10 embryos per well (*n* = 12 replicate wells for each 1:1 or environmentally relevant mixture). A 100% complete renewal of the solutions was performed daily for 8 days (1 mL until post hatch, after which volume was increased to 30 mL to account for difference in size). Mortality and signs of abnormal development were recorded daily throughout the exposure period. Fathead minnow embryos hatch begin hatching around 4 days post fertilization through 7- or 8-day post fertilization. In our laboratory, the majority of fish hatch by day 6 (55%–70%), so we evaluated effects on hatching rate on day 6. At the end of each exposure, total length (TL) of 2 representative fish from each exposure group was measured using ImageJ software (National Institute of Health and Laboratory for Optical and Computational Instrumentation, Madison, WI, United States) and used as an indicator of growth. Remaining fish were subsampled and used for behavioral assays as described below.

### 2.4 Potential for chronic exposure to mixtures of IM and TM to activate the nAChR *in vivo*

Behavioral indicators of nAChR activation were analyzed with two assays: embryonic motor activity following 1 day of exposure, and the predator escape response assessed after 8 days of exposure to the neonicotinoid mixtures. Video analysis was performed blind with respect to exposure concentration. The embryonic motor activity was assessed for all fish in an exposure group by quantifying the frequency of spontaneous contractions as described above for acute exposures.

The predator escape response was assessed in 1 representative fish from each exposure group according to the methods described in Painter et al. (2009) and McGee et al. (2009). A dissecting microscope was set up with a 1 by 1 mm grid attached to a stimulus plate with a vibrating buzzer in place of the glass base piece. A Phantom MiroC210 camera (Vision Research, Wayne, NJ, USA) was attached to the dissecting microscope to record videos at 1,000 fps. A ring light and a two-headed external light source were arranged on the microscope to increase visibility. Videos were recorded using the Phantom Camera Control Application software (Vision Research, Wayne, NJ, USA). The camera and the buzzer on the vibrational stimulus plate were activated simultaneously by a trigger. Individual larvae were subsampled from each replicate exposure group and placed in a plastic dish with 2 mL of standard culture water. This dish was placed on the vibrational stimulus plate and each larva was allowed 1 min to acclimate prior to the trigger for the camera and buzzer being activated and the predator escape response being recorded. In the rare occasion that a larva failed to produce a predator escape response after two stimuli attempts, a new larva from the same exposure group was selected (3 fish failed to respond across all exposure groups and replicate experiments). Videos were recorded in cycles for all experiments (1 larva for each replicate exposure group/concentration) to remove time as a source of variability. Video analysis was performed blind using the ImageJ software (National Institute of Health and Laboratory for Optical and Computational Instrumentation, Madison, WI, United States) following methods described by Diamond et al. (2016). Latency (amount of time between initial stimulus and initial response movement), burst speed (speed of initial 40 ms of response), and total escape response (combination of latency, length of larva, and burst speed) were measured from the videos.

### 2.5 Statistical analysis

Data analyses were conducted using SigmaStat (Ver 4 integrated with SigmaPlot11, SPSS Inc., Chicago, IL, United States). There were no significant differences in any endpoints among control groups; therefore all 1:1 and environmentally relevant mixtures were compared to the combined controls and across all mixture exposure groups. Survival data were analyzed using the Kaplan-Meier survival analysis with the Gehan-Breslow significance test. Remaining endpoints were analyzed in a single one-way analysis of variance (ANOVA) with Tukey’s *post hoc* tests at 95% confidence. Assumptions of normality and homoscedasticity were checked using Shapiro-Wilk and Levene’s tests, respectively. If data failed to pass normality, then a Kruskal–Wallis one-way ANOVA on Ranks with Dunn’s *post hoc* was used. Data are presented as the mean ± SEM. When a dose-response was not apparent, Chi Square analysis was used to determine whether the proportions of affected individuals (total percent within the entire treatment group) that had responses outside of control responses (values outside of the mean ±1 SD of control fish) were different from control.

## 3 Results

### 3.1 Effects of acute exposure to a mixture of IM and TM on behavioral indicators of nAChR activation

**FIGURE 1:**
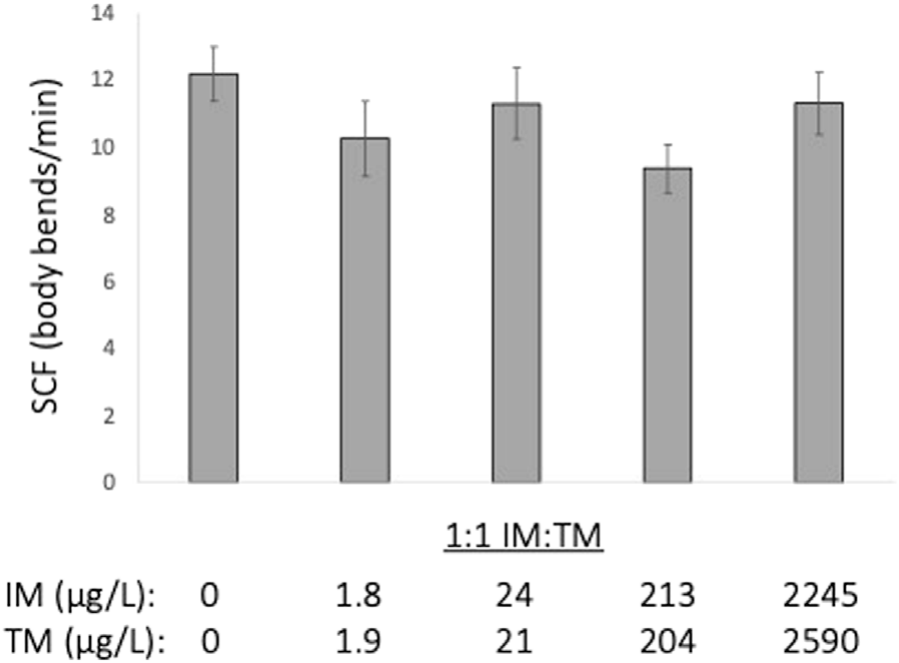
Acute (1 min) exposure to 1:1 mixture of imidacloprid (IM) and thiamethoxam (TM) does not alter embryonic motor activity of fathead minnow embryos (24 h post fertilization) (*p* = 0.548). Data presented are the mean ± SEM, *n* = 8 per treatment.

Acute exposure to a 1:1 mixture of IM and TM ranging from approximately 2–2,000 μg/L of each neonicotinoid did not alter embryonic motor activity (ANOVA, *p* = 0.548, Figure 1).

### 3.2 Chronic toxicity following exposure to mixtures of IM and TM

**FIGURE 2:**
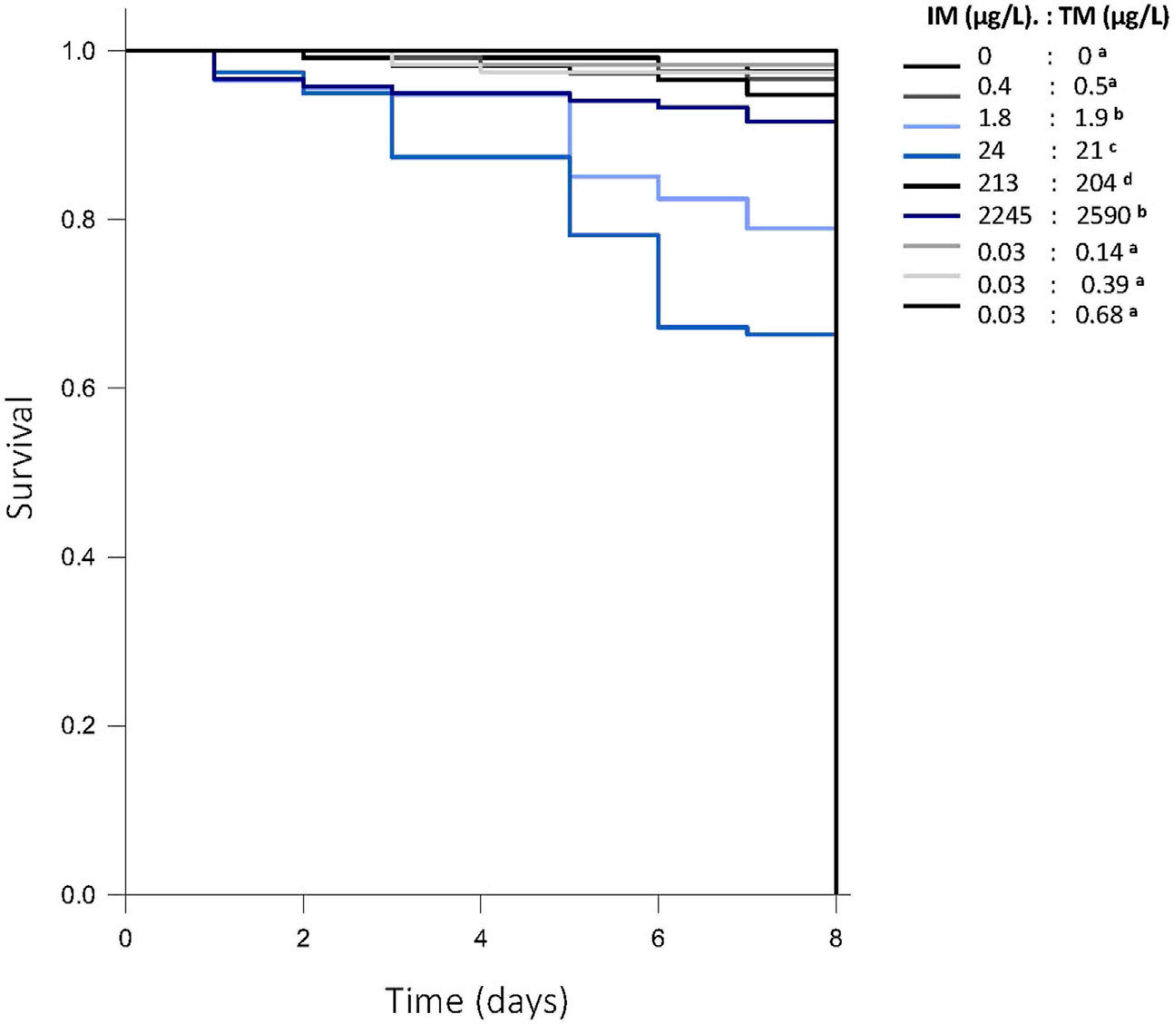
Survival of fathead minnow larvae following chronic exposure to various mixtures of imidacloprid (IM) and thiamethoxam (TM) (*p* < 0.001) from 1—8 days post fertilization. Survival lines that are in blue represent the treatment groups with lower survival than control (93% survival). Letters indicate significant differences between treatments. Data are presented as proportion fish surviving across all experiments, *n* = 120 fish/treatment.

Survival in control fish through 8 days post-fertilization was 93%. Chronic exposure to a 1:1 mixture of ∼2—2,000 μg/L of each neonicotinoid reduced survival by 5%–35% compared to control (*p* < 0.001), but not in a dose-dependent manner (Figure 2). Survival was not impacted following chronic exposure to mixtures at environmentally relevant ratios of IM and TM (Gehan-Brelow statistic = 179, df = 8, Figure 2). No overt signs of toxicity or developmental malformations were observed in any surviving fish.

**FIGURE 3:**
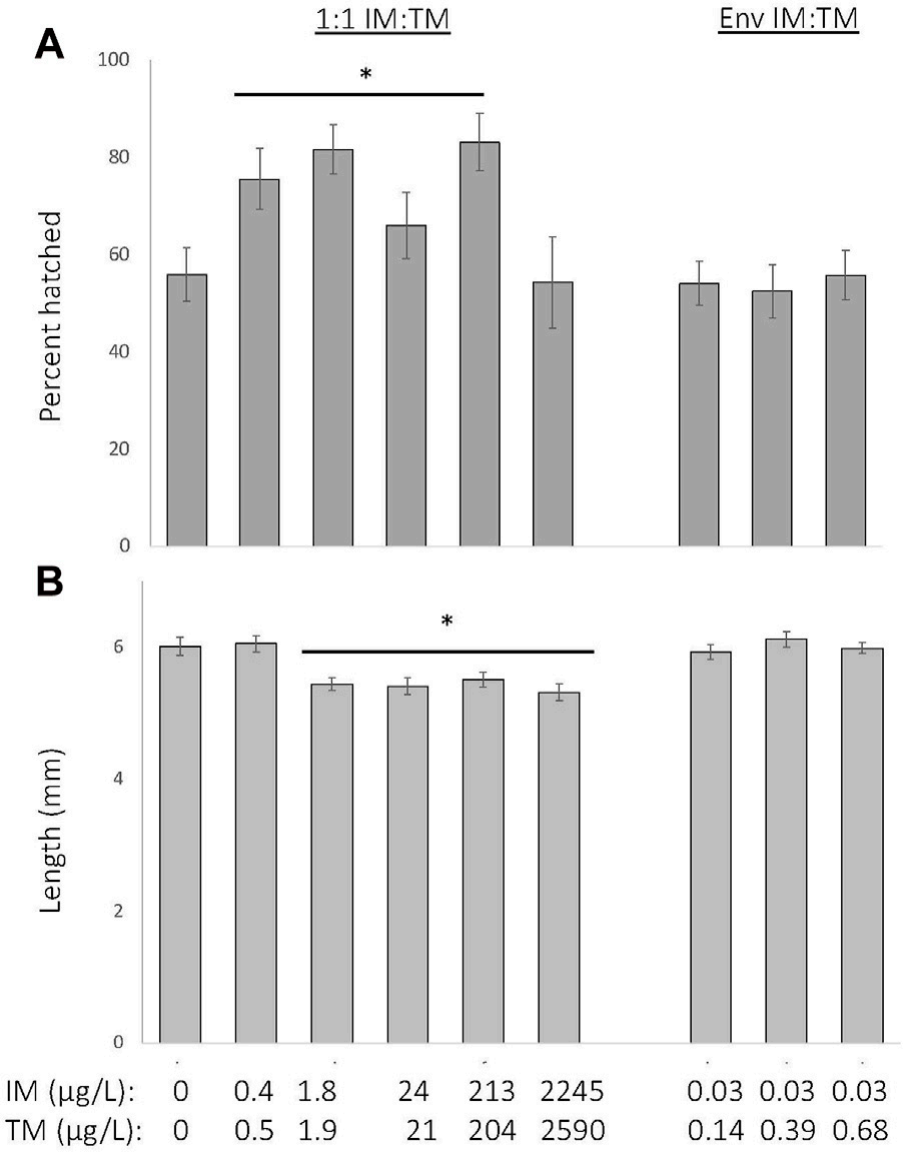
Sublethal effects of chronic exposure to various mixtures of imidacloprid (IM) and thiamethoxam (TM) on hatching success at 6 days post fertilization **(A)** and length at 8 days post fertilization **(B)**. * Indicates a treatment is significantly different from control (*p* < 0.001). Data are presented as mean ± SEM, *n* = 12 per treatment.

The percent of fish that hatched by 6 days increased by approximately 5%–20% following chronic exposure to a 1:1 mixture of ∼0.2—200 μg/L of IM and TM (*p* < 0.001), but was not significantly altered by exposure to environmentally-relevant ratio mixtures of IM and TM (Kruskal–Wallis H = 33, df = 8, Figure 3A). All control fish hatched by 8 days post-fertilization and all surviving fish exposed to all mixtures of IM and TM hatched by 8 days post-fertilization. At 8 days post-fertilization, fathead minnow larvae were 8%–11% smaller following chronic exposure to a 1:1 mixture of ∼2—2,000 μg/L of IM and TM (*p* < 0.001), but growth was not altered in larvae exposed to environmentally relevant ratio mixtures of IM and TM (ANOVA with Tukey *post hoc* test, df = (8, 182), F = 7.2, Figure 3B).

### 3.3 Effects of chronic exposure to mixtures of IM and TM on behavioral indicators of nAChR activation

**FIGURE 4:**
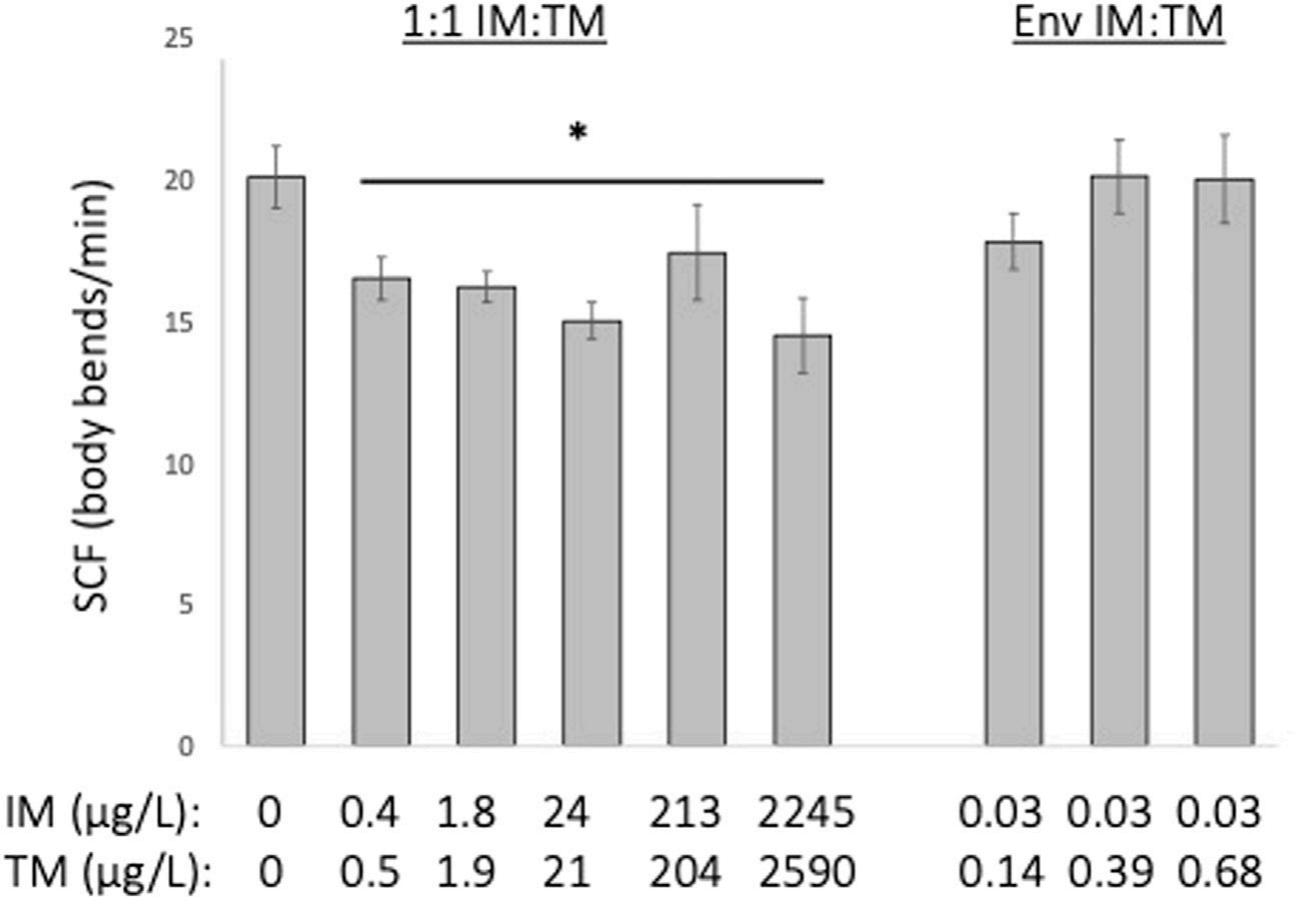
Behavioral indicators of nAChR activation following 24-h exposure to various mixtures of imidacloprid (IM) and thiamethoxam (TM). Embryonic motor activity is estimated using Spontaneous Contraction Frequency (SCF) of the tail in embryos that are 24 h old. *Indicates a treatment is significantly different from control (*p* = 0.04). Data are presented as mean ± SEM, *n* = 12 per treatment.

Embryonic motor activity of embryos exposed to a 1:1 mixture of ∼0.2—2,000 μg/L of IM and TM was reduced by approximately 18%–28% (*p* = 0.041) but was not altered following exposure to environmentally relevant ratio mixtures of IM and TM (Kruskal–Wallis H = 17.6, df = 8, Figure 4).

**FIGURE 5:**
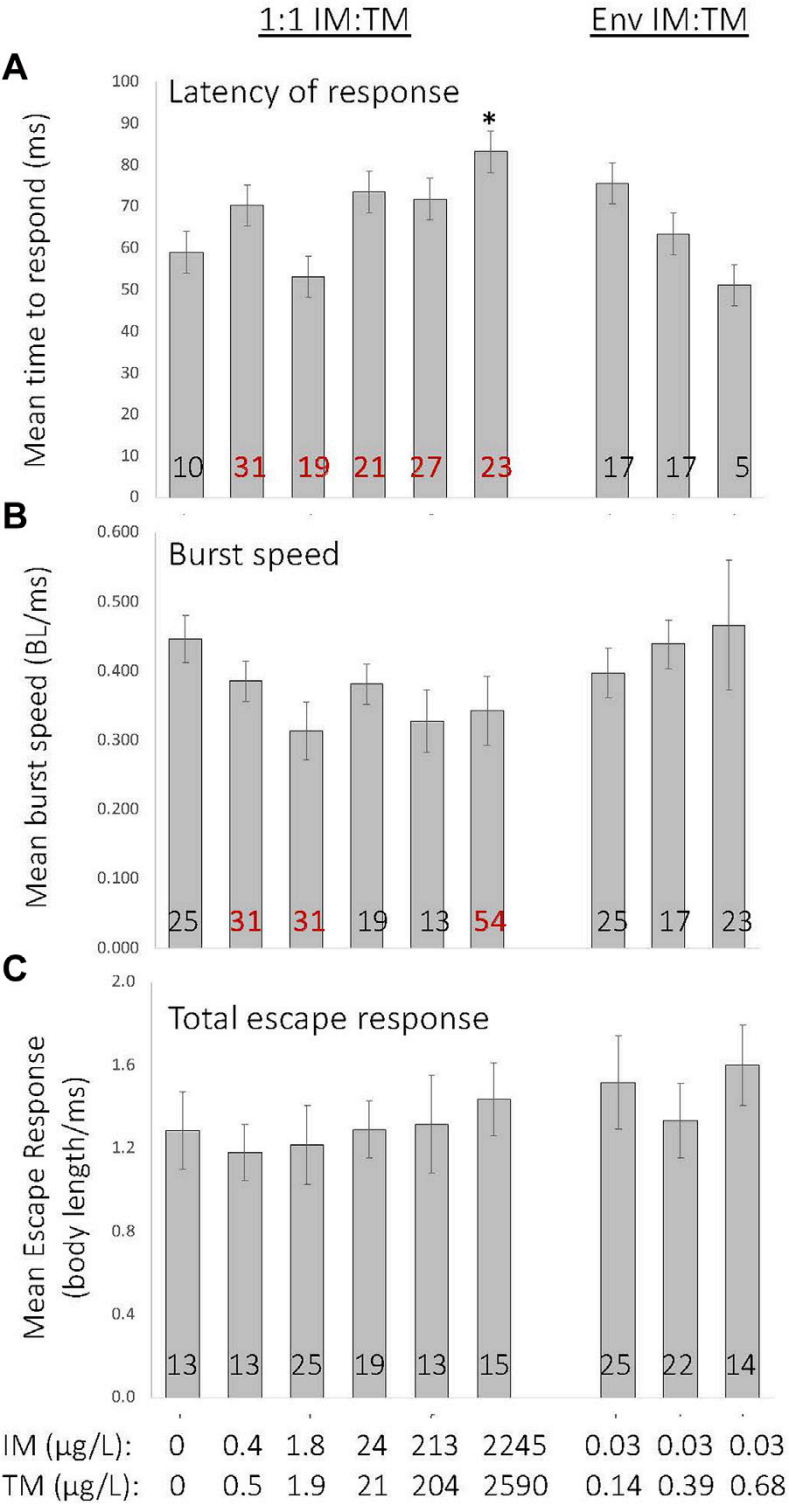
Effects of chronic embryo larval exposure to various mixtures of imidacloprid (IM) and thiamethoxam (TM) on the startle escape response in 8-day old fathead minnow larvae. Numbers within the bars indicate the proportion of individuals (as percent of total) that showed altered latency **(A)**, burst speed **(B)** or overall total escape response **(C)** compared with control. Those indicated in red were significantly different from control (*p* < 0.001). *Indicates a treatment is significantly different from control (*p* = 0.002). Data are presented as mean ± SEM, *n* = 12 per treatment.

Exposure to mixtures of IM and TM causes subtle alterations in the startle escape response. Chronic exposure to a 1:1 mixture of IM and TM at the highest ratio (∼2,000 μg/L of IM and TM) caused an increased latency in response to stimuli (*p* = 0.002, Kruskal–Wallis H = 23.9, df = 8, Figure 5A). A significant proportion of fish exposed to lower 1:1 mixtures were significantly slower to respond to stimuli (χ^2^ = 36.36, *p* < 0.001), but those exposed to environmentally relevant ratio mixtures were not slower to respond (Figure 5A). While a significant impact on burst swimming speed was identified by ANOVA on ranks (*p* = 0.044, Kruskal–Wallis H = 15.9, df = 8), a Dunn’s *post hoc* test failed to identify differences between treatment groups (Figure 5B). A significant proportion of fish exposed to 1:1 ratios of 0.2—2,000 μg/L of IM and TM had reduced burst swimming speed (*p* < 0.001), although a non-monotonic dose response was observed (Figure 5B, χ^2^ = 58.2; Figure 5B). Overall predator escape response does not appear to be altered following any mixtures of IM and TM (*p* = 0.06, Kruskal–Wallis H = 5.89 df = 8, *p* = 0.75; χ^2^ = 14.9, Figure 5C).

## 4 Discussion

Neonicotinoid insecticides have been detected in mixtures at concentrations at or above aquatic life benchmarks for invertebrates (US EPA, 2022) in freshwater systems across the United States (Morrissey et al., 2015a; Hladik and Kolpin, 2016; Senger, 2018; Wolfram et al., 2018). Further, after decades of use, these insecticides have been shown to be pseudopersistent within the environment, and pose a threat to ecosystem health (Goulson, 2013; Berens et al., 2021; Schmidt et al., 2022). Growing evidence indicates that while aquatic vertebrates are insensitive to overt toxic responses when exposed to environmentally relevant concentrations, chronic exposure may still pose a toxicological threat to aquatic vertebrates by impacting hatching and survival, growth, and other physiological and behavioral changes that could reduce overall health (Beggel et al., 2012; Gibbons et al., 2015; Robinson et al., 2017; Hong et al., 2018a; Thompson, 2020; Tian et al., 2020; Victoria et al., 2022a; Victoria et al., 2022b; Jeninga et al., 2023).

Since environmental exposure to pesticides predominantly occurs as mixtures (Hladik and Kolpin, 2016; DATCP, 2018; Berens et al., 2021), we sought to better understand the potential toxic responses of binary mixtures of the neonicotinoid pesticides IM and TM in fish. Despite that these insecticides typically exist in aquatic ecosystems as mixtures, information regarding the effects of mixtures of neonicotinoids and whether these mixtures act in an additive fashion are lacking for fish. To address this data gap, this study, which is an extension from our previous work, used a modified concentration-addition approach to study how chronic exposure to various binary mixtures of IM and TM at relatively low concentrations (to reflect environmental concentrations) affect relatively insensitive aquatic species like fish. Since we did not repeat exposures to individual IM or TM here, we cannot directly compare effects of binary mixtures of IM and TM to effects of individual neonicotinoids; however, since the exposure design was the same and included similar concentrations for each pesticide, we can make general comparisons. Caution should still be used in comparing these data sets as this mixture study included one experimental group exposed to concentrations 10-fold higher than that tested individually in the previous studies. Dose-responses were not observed when exposed individually or as mixtures (thus LC50s or EC50s cannot be calculated from this data) and the concentrations evaluated are well below published LC50 values for fish, toxic unit concepts or concentration-addition null models cannot be used to evaluate to discern whether mixtures of neonicotinoids cause additive toxicity in fish. While a toxic unit approach might conceptually help us understand whether these pesticides can act in additive fashion in fish, the concentrations required would likely be well above environmentally relevant concentrations, and therefore may be less useful in understanding environmental risks.

Our findings further demonstrate that mixtures of sublethal concentrations of neonicotinoids deviate from predicted additive toxic responses. Toxicity in fathead minnows following chronic exposure to varying mixtures of IM and TM appears to be driven by IM. These mixtures may increase toxicity compared to individual neonicotinoids, but responses are not likely to be additive or synergistic. Further, this work supports mounting evidence that there is an alternative mode of toxicity for neonicotinoids in fish that does not involve over-activation of the nAChR, and the importance of expanding toxicity studies to include mixtures of pesticides.

### 4.1 Chronic exposure to mixtures of IM and TM cause adverse health effects in fish

Both as mixtures and individually, IM and TM have been shown to reduce survival and growth in larval fishes when present at concentrations of 2–2,000 μg/L (Topal et al., 2017; Islam et al., 2019; Victoria et al., 2022b; Victoria et al., 2022a; Jeninga et al., 2023). While concentration-addition or response-additive modeling analyses would be required to discern mixture effects with certainty, a general comparison of results from this mixture study to our previously published results from studies in which fathead minnows were exposed to similar concentrations of IM and TM individually suggests that chronic exposure to binary mixtures of IM and TM may slightly increase mortality in fathead minnow larvae, but that substantial additive toxicity is not likely. In this study, chronic exposure to 1:1 mixtures increased mortality on average by 18%, compared to an average 10% increased mortality following exposure to IM alone (Jeninga et al., 2023), and an average 13% increased mortality following exposure to TM alone (Victoria et al., 2022a) alone. When increasing concentrations of TM are present with IM at concentrations below what has been shown to cause mortality in fathead minnow, no increased mortality was observed. This supports the idea that IM may be a stronger driver of toxicity when the two insecticides are found together. Individually, both IM and TM can cause overt toxicity in the mg/L range, which may result from DNA damage, oxidative stress, altered metabolism or disruption of hormones (Yan et al., 2016; Topal et al., 2017; Frew et al., 2018; Wolfram et al., 2018; Tian et al., 2020), but it is not known how chronic exposure to lower concentrations of IM and TM as mixtures leads to observed mortality. Chronic exposure to concentrations of neonicotinoids well below LC50 (lethal concentration for 50% of the individuals, or median lethal concentration) values may still reduce survival and cause other adverse health effects in fish. This indicates that chronic exposure may still pose a low risk to wild fish populations, despite their relative insensitivity to these insecticides and that risks are slightly increased when exposed to these insecticides as mixtures.

A diverse set of environmental contaminants spanning various modes of action have been shown to impair early growth in fish; hence, growth is an important apical endpoint with respect to informing risk assessments for various aquatic contaminants. Embryonic exposure to TM has only been shown to reduce growth in zebrafish (Victoria et al., 2022b), while embryonic exposure to IM has been shown to impair growth in larval carp, medaka, and zebrafish (Islam et al., 2019; Vignet et al., 2019). Chronic exposure to mixtures of IM and TM also reduce growth in larval fathead minnows, but the mixtures do not appear to cause an additive effect; rather, effects seem to align with those observed following exposure to IM alone. Fathead minnows exposed to mixtures had similar impacts on growth as seen following exposure to similar concentrations of IM alone (Jeninga et al., 2023), while chronic exposure to similar concentrations of TM did not impair growth in fathead minnows (Victoria et al., 2022a).

Similarly, while hatching was not altered by exposure to TM alone in either fathead minnow or zebrafish embryos (Victoria et al., 2022a; Victoria et al., 2022b), mixtures of IM and TM seem to induce hatching of fathead minnow larvae as was seen following exposure to IM alone in fathead minnow larvae (Jeninga et al., 2023). This further supports the idea that in mixtures of IM and TM, observed effects may be driven by IM and not increased when TM is also present. The timing at which hatch occurs plays a large role in survival in the first year for fish in natural environments (Divino and Tonn, 2007; Moravek and Martin, 2011). Changes in sensitive behavioral endpoints following exposure to mixtures in comparison to exposure to IM or TM alone also follow this pattern. A 1:1 mixture may cause slightly more disruption of these behaviors; however, observations align more with toxicity caused by IM (Jeninga et al., 2023), than TM (Victoria et al., 2022a). These results are discussed further with respect to potential mode of action below.

It remains uncertain how mixtures of neonicotinoids can impact aquatic species. Some studies in invertebrates indicate mixtures of neonicotinoids can cause additive toxicity, but antagonism and synergism have also been observed and depend on which neonicotinoids are present and at what concentrations (Maloney et al., 2017; Maloney et al., 2018). In general, the number of chemicals in a mixture can determine effects (Deneer, 2000; Lanteigne et al., 2015; Maloney et al., 2017; Maloney et al., 2018) Our findings suggest that mixtures of neonicotinoids at concentrations well below published LC50s for fish do not appear to cause additive toxic responses in fish, at least not when a generalized concentration addition model approach to evaluating the responses is followed (Howard and Webster, 2009). Similarly, when fish are exposed to a binary mixture of thiamethoxam and acetamiprid, thiamethoxam is more toxic than acetamiprid with respect to physiological responses, and exposure to binary mixtures of the two neonicotinoids fails to cause increase toxicity in fish (Veedu et al., 2022). Our findings also support findings from mixture studies in aquatic insects, where toxic effects of neonicotinoid mixtures in mayfly nymphs following exposure to clothianidin and thiamethoxam were amplified, but not necessarily additive, and driven mostly by the toxicity of imidacloprid (Macaulay et al., 2021). A mesocosm study paired with laboratory assays demonstrates that mixtures of imidacloprid and clothianidin caused nonadditive disruptions of trophodynamics and community structure, but that toxicity was dominated by imidacloprid (Schmidt et al., 2022). In this study, the small increase in mortality in combination with other adverse responses like changes in hatching and decreased growth suggest that even when co-occurring neonicotinoids are present in the environment below benchmark values, their presence could impact recruitment success of wild fish populations. Continued monitoring of these insecticides in the aquatic environment and further study of sublethal toxic responses in aquatic vertebrates is warranted.

### 4.2 Is the toxicity of neonicotinoids mediated by the nAChR in fish?

While both IM and TM have low affinity for the nAChR in vertebrates (Matsuda et al., 2005; Ensley, 2018), both have been shown to alter sensorimotor functions, potentially due to altered cholinergic signaling (Crosby et al., 2015; Liu et al., 2018; Vignet et al., 2019; Tian et al., 2020; Victoria et al., 2022a; Victoria et al., 2022b; Jeninga et al., 2023). Here we use two behavioral endpoints, embryonic motor activity and the startle escape response, as an indicator of the potential for these low affinity nAChR agonists to interfere with normal cholinergic signaling. There are no published reports regarding the effects of exposure to mixtures of neonicotinoids on neurobehavioral endpoints in fish. Chronic exposure to ≥ 0.16 μg/L of IM alone or to high concentrations of TM alone (155 μg/L) cause an increase embryonic motor activity in fathead minnow larvae (Victoria et al., 2022a; Jeninga et al., 2023). Here, we show that when both IM and TM are present together, chronic exposure to a 1:1 mixture of IM and TM suppresses the embryonic motor response (Figure 4). Dose-dependent hyper-activity at lower concentrations leading to complete paralysis of embryonic motor activity following exposure to higher concentrations has been observed in fish larvae following exposure to strong nAChR agonists such as nicotine, amphetamines, cocaine, and some pesticides (Thomas et al., 2009; Tierney, 2011; Victoria et al., 2022b). Our data would then suggest that as mixtures, the presence of IM and TM together may overstimulate the nAChR causing hyperactivity, but not sufficiently interfere with cholinergic signaling to the extent that would lead to paralysis. Given the nature of our experimental design and the lack of a dose-response, we cannot determine whether IM and TM are acting in an additive fashion.

While the startle escape response is a more complicated behavior, it can also be disrupted by nAChR agonists by increasing the latency of response and decreasing the burst speed resulting in an overall reduction in the total escape response (Victoria et al., 2022b). While a significant proportion of fathead minnow larvae are slower to respond to stimuli and have reduced swimming speed when attempting to escape a predatory signal when exposed to IM or TM alone, overall these aspects of the response were not significantly altered (Victoria et al., 2022a; Victoria et al., 2022b; Jeninga et al., 2023). Here we show that chronic exposure to a 1:1 mixture of significantly greater concentrations of IM and TM (∼2,000 μg/L of each insecticide) is capable of significantly increasing the latency of response (Figure 5A), exposure to lower concentrations of a 1:1 mixture IM and TM only impacted latency of response and burst speed in a proportion of larvae tested. In comparison to effects following exposure to each insecticide alone, effects more closely resemble those of IM. As with our embryonic motor activity data, these impacts on the startle escape response suggest that when present together, IM and TM may have a greater interference with the cholinergic signaling required to regulate the startle escape response, but we cannot determine whether these effects are acting in an additive fashion.

Since there is a lack of a substantial disruption of behavioral response to exposure to these insecticides alone or in mixtures (e.g., leading to paralysis or significant latency or reduced speed in the startle response), it is possible that the impacts on these sensorimotor behaviors are not due to direct interaction with the nAChR, but rather some other aspect of regulation of these behaviors. These pesticides might cause toxicity through generalized neurotoxicity or through other modes of action all together. Zhang et al. (2023) showed that zebrafish exposed to similar concentrations of TM and IM (individually) had altered expression of several genes relevant to locomotor-related behaviors but not directly related to cholinergic signal cascades, as well as those associated with development of the nervous system. While predictive of causing alterations in locomotor behaviors, exposure to IM or TM does not appear to manifest in substantial alterations in the embryonic motor response or C-start locomotory behaviors, either individually (Victoria et al., 2022a; Victoria et al., 2022b; Jeninga et al., 2023) or in combination, as shown here. However, other locomotory behaviors altered by exposure to neonicotinoids have been reported (Crosby et al., 2015; Robinson et al., 2017; Liu et al., 2018) and neurotoxic potential has been established in several studies (Topal et al., 2017; Tian et al., 2020; Zhang et al., 2021); therefore, the neurotoxicity of IM and TM still warrants further study. Mounting evidence suggests that neonicotinoid insecticides may cause toxicity through other modes of action in vertebrates independent of neurotoxicity, including alteration of the hypothalamic-pituitary-gonad and thyroid axes (Zhu et al., 2019), general metabolism and corticosteroid pathways (Veedu et al., 2022; Zhang et al., 2023), generalized oxidative stress (Yan et al., 2016; Veedu et al., 2022), or by disrupting the immune system (Mason and Sample Organization, 2013; Morrissey et al., 2015b; Gibbons et al., 2015; Hong et al., 2018b; Kizilkaya et al., 2023). A better understanding of the sublethal responses to neonicotinoids and their impacts on non-target fish species would enable us to better monitor the impacts of agricultural insecticides on aquatic ecosystems.

### 4.3 Conclusion

This follow-up study from our previous work evaluating the toxicity of individual neonicotinoids in fish still brings us closer to understanding the potential risks that mixtures of neonicotinoid pesticides pose to fish, particularly since we assessed the toxicity of binary mixtures at concentrations that are similar to those detected in the environment. Differences in outcomes between single insecticide exposures and mixture exposures has important implications for toxicity testing, as standard practice for pesticide testing and regulation targets compounds individually, rather than in mixtures (Weisner et al., 2021). The pesticide registration process generally requires standardized toxicity test protocols be followed, but there are currently no standardized test protocols for chemical mixtures. Regulation of pesticides, once registered, also targets compounds individually rather than in mixtures (Weisner et al., 2021). There are no water quality criteria or aquatic life benchmarks for pesticide mixtures, either. Because pesticides are detected as mixtures in the environment (Hladik and Kolpin, 2016; Senger, 2018; Wolfram et al., 2018; Schmidt et al., 2022), more study of pesticide mixtures is needed before we can accurately predict ecological risks (Weisner et al., 2021). Therefore, when assessing the toxicity of IM and TM in the laboratory and in natural habitat settings, it is beneficial to consider how they may behave in a mixture.

This work investigated the possible toxicological risks posed by binary mixtures of two neonicotinoids, IM and TM, to a model organism, the fathead minnow. These mixtures significantly impacted hatching, growth, and survival at and above environmentally-relevant concentrations of individual IM or TM, but not at environmentally relevant mixtures of IM and TM in Wisconsin surface waters. While other physiological and behavioral endpoints were not substantially impacted, our findings that 1:1 mixtures have a greater impact on embryonic motor activity and the startle escape response then either IM or TM alone raise questions about how sublethal concentrations of a mixture of neonicotinoids may impact fish survival in the environment, as well as questions about other modes of action for neonicotinoids in fish. In summary, continued study of the effects of IM and TM individually and as mixtures is necessary, as is a to determination of the mode of action of neonicotinoids for observed toxicity in fish.

## Data Availability

The original contributions presented in the study are included in the article/Supplementary material, further inquiries can be directed to the corresponding author.
